# Multivalent Interactions Between the Picornavirus 3C(D) Main Protease and RNA Oligonucleotides Induce Liquid–Liquid Phase Separation

**DOI:** 10.3390/v17111473

**Published:** 2025-11-04

**Authors:** Somnath Mondal, Saumyak Mukherjee, Kevin E. W. Namitz, Neela H. Yennawar, David D. Boehr

**Affiliations:** 1 Department of Chemistry, The Pennsylvania State University, University Park, PA 16801, USA; sjm7540@psu.edu (S.M.);; 2 Department of Theoretical Biophysics, Max Planck Institute of Biophysics, 60438 Frankfurt am Main, Germany; saumyak.mukherjee@biophys.mpg.de; 3 The Huck Institutes of the Life Sciences, The Pennsylvania State University, University Park, PA 16801, USA

**Keywords:** 3C protease, protein–RNA interactions, NMR spectroscopy, liquid–liquid phase separation, picornavirus

## Abstract

The picornavirus 3CD protein is a precursor to the 3C main protease and the 3D RNA-dependent RNA polymerase. In addition to its functions in proteolytic processing of the virus polyprotein and cleavage of key host factors, the 3C domain interacts with cis-acting replication elements (CREs) within the viral genome to regulate replication and translation events. We investigated the molecular determinants of RNA binding to 3C using a wide range of biophysical and computational methods. These studies showed that 3C binds to a broad spectrum of RNA oligonucleotides, displaying minimal sequence and structure dependence, at least for these shorter RNAs. However, they also uncovered a novel aspect of these interactions, that is, 3C-RNA binding can induce liquid–liquid phase separation (LLPS), with 3CD–RNA interactions likewise leading to LLPS. This may be a general phenomenon for other 3C and 3C-like proteases and polyproteins incorporating 3C domains. These findings have potential implications in understanding virally induced apoptosis and the control of stress granules, which involve LLPS and include other proteins with known interactions with 3C/3CD.

## 1. Introduction

Positive-strand RNA viruses generate a polyprotein that must then be proteolytically processed into the components necessary for virus replication and packaging [1,2]. These viruses have evolved strategies to maximize their genomic information content. For example, intermediates in the proteolytic processing pathway may have different and/or emergent functions compared to their fully processed counterparts. In addition, many viral proteins have multiple functions. These ideas are exemplified by the picornavirus 3CD, 3C, and 3D proteins [3,4,5]. 3CD comprises domains encompassing the 3C protease and the 3D RNA-dependent RNA polymerase [3,4,6]. The 3CD and/or 3C proteases cleave the viral polyprotein and host cell defense proteins targeting viral RNA synthesis and replication [7,8,9]. The 3C domain interacts with viral RNA replication elements, including oriL, oriI, and oriR, to regulate viral replication and translation, thereby coordinating the viral life cycle [10,11,12,13]. The molecular determinants governing 3C–RNA interactions and how these interactions may affect other 3C/3CD functions are poorly understood.

3C and 3C-like proteases are found in a wide range of positive-strand RNA viruses, particularly picornaviruses (poliovirus, PV; human rhinovirus, HRV; hepatitis A virus, HAV; foot-and-mouth disease virus, FMDV; coxsackievirus B, CVB; cardio virus; enterovirus-D68, EV-D68; and enterovirus 71, EV71), coronaviruses (severe acute respiratory syndrome coronavirus 1 and 2, SARS-CoV and SARS-CoV-2; Middle East respiratory syndrome coronavirus, MERS-CoV), alphaviruses (Chikungunya virus, eastern equine encephalitis virus), flaviviruses (dengue virus, zika virus, West Nile virus, yellow fever virus), caliciviruses (norovirus, Sapo virus), and astroviruses [11,14,15,16]. The 3C three-dimensional structures are highly conserved, consisting of two antiparallel, six-stranded β-barrel domains, and catalytic mechanisms generally identical, resembling those of trypsin-like Ser proteases but with a Cys-nucleophile [3,4,11]. In PV, the 3C catalytic triad consists of His40, Glu71, and Cys147, which are spatially arranged to facilitate the catalytic reaction.

The 3C protein and/or the 3C domain in 3CD also have important RNA-binding capabilities. For example, 3CD interacts with the stem-loop d of oriL, enhancing the binding of the poly(rC)-binding protein 2 (PCBP2) to the stem-loop b [7,8,17]. Additionally, the RNA-binding abilities of both 3C and 3CD through oriI are indispensable for the effective uridylylation of the VPg protein [18]. Previous studies have identified the 80’s region of 3C as highly important for binding to the stem-loop of oriI [18]. Given the sequence/structural diversity of these RNA elements, it is unclear what factors determine these interactions. A fuller understanding of these interactions may provide new insights into the development of anti-viral therapeutics that disrupt these crucial interactions.

To better define the molecular determinants of RNA interactions with PV-3C and PV-3CD, we assessed the binding of a variety of RNA oligonucleotides with diverse sequences and secondary structure capabilities using a range of experimental and computational methods. While little specificity was found among this set of RNA oligonucleotides, these interactions surprisingly lead to higher-order complex formation and liquid–liquid phase separation (LLPS) for both PV-3C and PV-3CD. LLPS has previously been found in various viral infections, and it may play a role in virally induced apoptosis and virus dissemination through the formation/deformation of stress granules [19,20,21]. As such, these findings provide a potential new role for 3C/3CD in these processes and potentially reveal another level of regulatory processes involving these proteins.

## 2. Materials and Methods

### 2.1. Materials

^15^N-ammonium chloride (NH_4_Cl) and D2O (D, 99.9%) were purchased from Cambridge Isotope Laboratories (Andover, MA, USA). All reagents were bought from VWR (Radnor, PA, USA) unless otherwise specified. The IS307 iSpacer with a depth of 0.3 mm, was purchased from SunJin Lab Co. (Hsinchu City, Taiwan) for use in Differential Interference Contrast (DIC) microscopy.

### 2.2. Protein Expression and Isotopic Labeling

Plasmids (pSUMO) encoding C-terminal hexa-histidine-tagged PV 3C, 3D, and 3CD were transformed into Escherichia coli BL21(DE3) pRARE cells. In 3D and the 3D domain of 3CD, the following amino acid substitutions were present to avoid aggregation (compared to type 1 Mahoney strain): L446D and R455D [4,22]. On 3C and in the 3C domain of the 3CD, the following amino acid substitutions were included to inhibit aggregation and disrupt the intermolecular cleavage of 3CD: E55A, D58A, E63A, and C147A. These aggregation-preventing mutations hinder oligomerization interfaces on 3C and 3D, enabling large concentrations of 3CD required for NMR and other biophysical studies.

Following transformation, cell cultures were shaken in 10 mL M9 minimal media (6.0 g/L Na_2_HPO_4_, 3.0 g/L KH_2_PO_4_, 0.5 g/L NaCl, 2 g/L glucose, 1.0 g/L NH_4_Cl, 0.1 mM CaCl_2_, 1 mM MgSO_4_, trace metal mix (Teknova, Hollister, CA, USA), MEM vitamin mix (Thermo Fisher, Bellefonte, PA, USA), 50 g/mL kanamycin, and 30 g/mL chloramphenicol) for 16–20 h until the optical density at 600 nm (OD600) was higher than 0.6. For ^15^N labeling, 1 mL of the 10 mL culture was inoculated to 50 mL of M9 medium and shaken overnight at 200 rpm at 30 °C. The next morning, 20 mL of the overnight culture was used to inoculate 1 L of M9 media (including 1 g/L ^15^N NH_4_Cl from Cambridge Isotope Laboratories, 2 mM MgSO_4_, 12.0 g/L Na_2_HPO_4_, 6.0 g/L KH_2_PO_4_). Protein expression was initiated with 1 mM isopropyl-D-1-thiogalactopyranoside (IPTG) once OD600 reached 0.6–0.8, and the cultures were then shaken at 25 °C. After 16–20 h, cells were harvested through centrifugation (3900× *g*, 30 min, 4 °C), rinsed with buffer (10 mM Tris, 1 mM EDTA pH 8), and centrifuged again (3800× *g*, 15 min, 4 °C). Solutions were then decanted and the resultant pellets weighed before storing at −80 °C. Unless otherwise mentioned, all the cultures were incubated at 37 °C while shaking at 200–250 rpm.

### 2.3. RNA Sample Preparation

All the RNA oligonucleotides (RNA-1–RNA-15) were purchased from Horizon Discovery Biosciences LTD (Cambridge, UK) (Figure S1 and Table S1) (USA) with standard HPLC purification and desalting. The RNA samples were dissolved in a buffer containing 10 mM HEPES pH 7.5 and 50 mM NaCl to prepare 1 mM stock, which were subsequently diluted to the required working concentrations for each biophysical experiment.

The RNA oligonucleotides (RNA-1 to RNA-15) were selected to encompass viral RNA replication elements and structurally related motifs across a broad phylogenetic spectrum, primarily from enteroviral genomes (oriL and oriR of poliovirus and related enteroviruses), supplemented with non-picornaviral and synthetic RNA controls (like poly(A), poly(U), and SARS-CoV-2-derived sequence). This selection ensured the representation of both conserved and divergent RNA elements to assess the generality of 3C–RNA recognition.

### 2.4. Purification of PV-3C and PV-3CD

Cell pellets with expressed PV-3C were resuspended in 20 mM HEPES, 50 mM NaCl, 1 mM EDTA, 5 mM β-mercaptoethanol, 5 mM imidazole, pH 7.5, with 1.4 g/mL pepstatin A and 1 g/mL leupeptin. Cell pellets that expressed PV-3CD were resuspended in a buffer containing 100 mM potassium phosphate, 10 mM β-mercaptoethanol, 120 µM ZnCl_2_, 20% glycerol, pH 8.0, with 1.4 g/mL pepstatin A, 1 g/mL leupeptin, and 500 mM phenylmethanesulfonyl fluoride (PMSF). The cells were then lysed, followed by polyethyleneimine (PEI) precipitation and ammonium sulfate precipitation to 60% saturation. The ammonium sulfate pellet was resuspended, followed by the purification of proteins by nickel–nitrilotriacetic acid (Ni-NTA) affinity chromatography, following protocols outlined in refs [4,22,23]. Following the cleavage of the SUMO tag by 1–2 g ubiquitin-like-specific-protease (ULP-1), samples were dialyzed against 100 mM potassium phosphate (pH 8.0), 100 mM sodium chloride, 60 mM ZnCl_2_, 5 mM β-mercaptoethanol, and 20% glycerol. The samples were then concentrated using 30 kD molecular weight cutoff (3CD) or 10 kD molecular weight cutoff (3C) Sartorius Vivaspin spin concentrators in a buffer containing 10 mM HEPES and 50 mM NaCl at pH 7.50.

### 2.5. NMR Sample Preparation

To prepare ^15^N labeled protein samples, we utilized the buffer containing 10 mM HEPES pH 7.5 and 50 mM NaCl with 10% D2O for deuterium lock. The protein samples were concentrated using a 0.5 mL Millipore Amicon Ultra Centrifugal Filters, 3K MWCO (Sigma-Aldrich, St. Louis, MO, USA). The protein concentration was calculated by measuring the absorbance at 280 nm (the molar extinction coefficient (ε) for 3C = 8960 M^−1^ cm^−1^, 3CD = 84,690 M^−1^ cm^−1^). All NMR experiments were carried out on a 600 MHz Bruker NEO spectrometer with 5 mm TCI single-axis gradient cryoprobes (Bruker, Billerica, MA, USA). NMR data was processed using NMRFAM-SPARKY software [24]. The ^1^H-^15^N SOFAST-heteronuclear multiple quantum coherence (HMQC) spectra were run with 32 ns, 128 points were used in the indirect dimension (F1), and 2048 points were used in the direct dimension (F2) [25,26,27,28,29].

### 2.6. Sedimentation Velocity Analytical Ultracentrifugation (AUC)

PV-3C and PV-3CD, both with and without RNA-1 and/or RNA-2, were loaded into 3 mm Epon-charcoal centerpieces sandwiched between sapphire windows for analytical ultracentrifugation analysis [30]. The cells were loaded into either an An50 or An60 titanium rotor (depending on number of samples for the run), and the rotor was then placed in the vacuum chamber of an Optima multiwavelength analytical ultracentrifuge (Beckman Coulter Life Sciences, Brea, CA, USA). The vacuum chamber was evacuated, and then the rotor and samples were allowed to equilibrate to the experimental temperature of 25 °C for ~2 h. Once equilibrated, a method was written for the run in the UltraScan III software 61. For this method, the rotor accelerated to 42,000 RPM, and radial scans of each sector were performed every 2 min for 16 h. For AUC measurements, the wavelength used for 3C was 280 nm and 290 nm for 3CD. A wavelength of 280 nm is most used for protein detection in AUC due to the presence of aromatic amino acids. Therefore, we chose a wavelength of 280 nm for the AUC measurement of 3C (MW = 20.69 kDa), whereas the signal was too high for 3CD at this wavelength because 3CD (MW = 71.92 kDa) is a larger protein, resulting in higher absorption. As a result, we picked 290 nm for the AUC measurement of 3CD to keep the absorbance at or below 1. Once the run was completed, the data were analyzed using UltraScan III. The data were first converted from raw radial intensity data to pseudo-absorbance data. The scans were then fit to solutions of the Lamm equation, with additional fitting to account for time-independent noise. Once the RMSD for the fits were <0.003, the data were re-fit to determine the correct meniscus position for each sample and to account for radially invariant noise as well. Then, a final iterative fit was performed. The final S-value range was 1–10 with a resolution of 100, and the final frictional ratio range was 1–4 with a resolution of 64.

### 2.7. Differential Interference Contrast (DIC) Microscopy

DIC microscopy was performed utilizing an Olympus BX 61 microscope (Evident Scientific, Waltham, MA, USA) at the Microscopy Core Facility (Huck), Pennsylvania State University. We utilized the biological configuration with automated four-color plus DIC image collection, polarization, darkfield, and brightfield [31,32,33]. The illumination happens through mercury vapor, provided through either a rapid automated shutter or conventional filter cubes. The objective of the Biological Configuration includes UplanFL 40×/0.75 for our images. All objectives in this microscope have DIC optics. The digital cameras were Hamamatsu cooled digital cameras (ORCA ER, Model C4742-80) and Olympus DP71 (Evident Scientific, Waltham, MA, USA). The prior controller with the joystick controlled focus and location. CellSens software (version 4.3) was used for image processing. PV-3C and PV-3CD samples with RNA in the HEPES buffer (10 mM HEPES pH 7.5 and 50 mM NaCl) were prepared, and a drop was mounted on clean glass slides with a coverslip for high-quality DIC microscopy imaging. There was no staining, and appropriate controls of buffer, protein, and RNA were recorded before observing LLPS in the protein–RNA samples.

### 2.8. Negative Staining Electron Microscopy

For visualization by transmission electron microscopy (TEM), the samples were subjected to negative staining. A 3.5 μL aliquot of the sample was applied to a carbon support TEM grid (400 mesh, Ted Pella, Redding, CA, USA) and incubated for 1 min [34]. Subsequently, excess liquid was carefully blotted away using filter paper. The grid was then washed twice with 10 μL of ultrapure water to remove any unbound material. The grid was stained with 10 μL of freshly prepared uranyl formate solution (0.7% *w*/*v*, pH 4.5) for 30 s. Finally, after blotting away excess staining solution, the grid was air-dried at room temperature.

### 2.9. Molecular Dynamics (MD) Simulation: System Preparation

**Figure 1:**
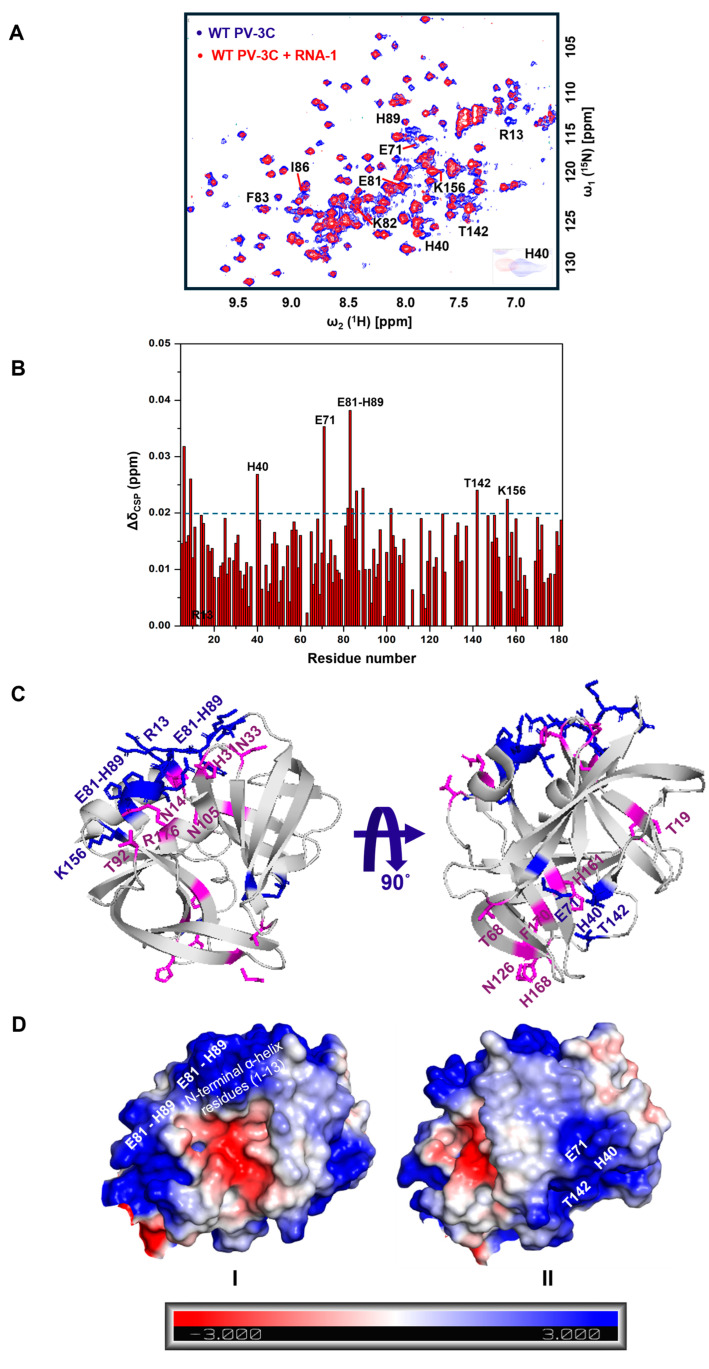
RNA binding leads to chemical shift changes for two oppositely faced residue clusters in PV-3C. (**A**) ^1^H-^15^N SOFAST-HMQC NMR spectra were compared with free wild-type PV-3C (blue) and RNA-1 (GGCGGCGUACUCCGG)-bound PV-3C (red) in 1:0.5 stoichiometry. Residues with substantial chemical shift perturbation (CSP) are labeled in the spectra; zoomed-in resonances represent CSP change for the corresponding residues. (**B**) NMR CSPs for each residue is plotted, with residues labeled that show a considerable change in CSP after RNA-1 addition. Note that the R13 peak was missing after the addition of RNA-1. NMR CSPs were calculated using the following equation: Δδ_combined_ = (ΔδH^2^ + (ΔδN/5)^2^)^0.5^, where Δδ_H_ and Δδ_N_ are the chemical shift differences between 3C with and without RNA for the backbone amide proton and nitrogen, respectively. Resonances with substantial CSPs have Δδ_combined_ greater than 0.02 ppm (blue dashed line). NMR assignment of 3C is depicted from BioMagResBank database (https://bmrb.io/ (accessed on 28 October 2025)) under BMRB accession number 15222, by Amero CD et al. [14]. (**C**) Residues showing substantial CSPs in the presence of RNA are represented in blue on the PV-3C X-ray crystal structure (PDB ID: 1L1N). Pink residues represent substantial CSPs in the presence of RNA-3-RNA-15 overall on PV-3C (see Figures S2–S14). The PV-3C protein concentration was 25 µM and RNA was added following a 1:0.5 stoichiometry. Both protein and RNA were in a buffer containing 10 mM HEPES, pH 7.5, and 50 mM NaCl. The ^1^H-^15^N SOFAST-HMQC NMR experiment was recorded in a 600 MHz Bruker NEO spectrometer equipped with z-gradient triple-resonance (^1^H, ^13^C, ^15^N) TCI cryogenic probe at 298 K. (**D**) The electrostatic surface potential map for PV-3C highlights Cluster-I and Cluster-II regions, with blue and red representing positively and negatively charged regions, respectively.

The initial coordinates of the PV-3C and PV-3CD proteins were obtained from the Protein Data Bank (PDB: 1L1N and 2IJD, respectively) [6,11,35]. The last three residues (Gln181, Ser182, and Gln183) were missing from the crystallographic PDB structure of 3C. As such, these were modeled using Pymol-2.5.0 and refined using the ModLoop web server of Modeler [36,37,38]. The initial structures of the RNA fragments of RNA-1 (GGC GGC GUA CUC CGG) and RNA-2 (CAU ACU GUU GUA GGG GAA) were generated using the NAflex web server [39]. Two chains of each RNA were put in a cubic box of sides ~11 nm and ~15 nm, along with PV-3C and PV-3CD, respectively, resulting in a total of four protein–RNA systems. The cluster-I region (see Figure 1) readily interacts with the RNAs, whereas the cluster-II region (see Figure 1) shows lower RNA-binding propensity. Hence, one of the RNA chains was randomly placed in the simulation box, whereas the second chain was placed in the vicinity of cluster-II. Each system was solvated with a three-point water model. Approximately 50,000 and 100,000 water molecules were added to the systems containing PV-3C and PV-3CD, respectively. Sodium ions were added to neutralize the systems. Salt at 50 mM NaCl was added to mimic experimental conditions. The protein, RNA, and ions were modeled using the CHARMM36m force-field, and the CHARMM-modified TIP3P model was used to describe the water molecules [40].

### 2.10. MD Simulation Runs and Analysis

All MD simulations were performed using the GROMACS-2022.4 simulation package [41]. The following simulation protocol was applied to both PV-3C and PV-3CD systems. An initial energy minimization was performed using the steepest descent algorithm to remove unrealistic atomic contacts. This was followed by a short 1 ns equilibration under NpT conditions (temperature (T) = 300 K and pressure (p) = 1 bar) with harmonic position restraints on the heavy atoms of the protein and the RNA (with a force constant of 1000 kJ mol^−1^ nm^−2^) to let the water and ions equilibrate. Thereafter, the position restraints were lifted, and the system was allowed to equilibrate under NVT conditions (T = 300 K) for 10 ns. This was followed by a 500 ns production run under similar NVT conditions, with the data being saved at a frequency of 10 ps.

All atomistic MD simulations were performed using the leap-frog integrator with a time step of 2 fs. The v-rescale (stochastic velocity rescaling) thermostat and the c-rescale (stochastic exponential relaxation) barostat were used to control temperature and pressure (wherever necessary), respectively [42]. Protein bonds involving hydrogen atoms and the internal degrees of water molecules were constrained using the LINCS and SETTLE algorithms, respectively [43]. Short-range electrostatic and Lennard-Jones interactions were calculated up to a distance of 1.0 nm. Long-range electrostatic interactions were treated with the particle-mesh Ewald technique with a grid spacing of 0.12 nm [44].

## 3. Results

### 3.1. PV-3C Binds to a Diverse Set of RNA Oligonucleotides

To better understand the molecular determinants of RNA binding to 3C, we collected NMR spectra for PV-3C in the presence and absence of 15 sequence- and structure-divergent RNA oligonucleotides (Figure 1 and Table S1). Some of these RNA oligonucleotides were based on sequences derived from oriL and/or oriR (e.g., RNA-1, 5′-GGCGGCGUACUCCGG-3′ from oriL in Figure 1 and Figure S1). Here, we focus on NMR spectra with RNA-1, but, notably, similar results were obtained for all RNA tested from RNA-2 to RNA-15 (Figures S2–S14), suggesting little to no sequence dependence of RNA binding to 3C using this set of RNA oligonucleotides.

[^15^N,^1^H] SOFAST-HMQC NMR spectra were collected after titrating ^15^N-labeled PV-3C with increasing concentrations of RNA-1. The addition of RNA (shown in Figure 1) led to chemical shift perturbations (CSPs) for ^1^H-^15^N backbone amide resonances belonging to amino acid residues in the previously identified RNA-binding region (E81–H89), the N-terminal α-helix residues (1–13), the active site residues (H40 and E71, part of the catalytic triad), and nearby residues T142 and K156 (Figure 1A,B). These amino acid residues appear to belong to two clusters (Figure 1C): Cluster-I and Cluster-II. Cluster-I is located near the KFRDI motif, which has been linked to RNA binding in earlier NMR investigations and mutational studies [4,14]. The N-terminal h1 helix is near this RNA-binding region and has also been previously implicated in RNA binding [11,14]. Recent reports indicate that Coxsackievirus 3C protein interacts with cloverleaf stem-loop D RNA through the KFRDI motif, the N-terminal h1-helix, and K156 residues, which is consistent with Cluster-I residues observed in our NMR studies. Cluster-II is on the opposite side of the protein, including the catalytic and adjacent residues (Figure 1C) [45,46]. It is noted that we took into consideration the results for all the different RNA oligonucleotides (also see Figures S2–S14) when identifying these two clusters. Intriguingly, mapping of electrostatic surface potentials indicates that both Cluster-I and Cluster-II regions are positively charged regions, suggesting a mechanism by which both Clusters could interact with negatively charged RNA (Figure 1D).

It is noted that the CSPs are smaller but are in the same order of magnitude to similar studies [14,47]. A challenge of these studies was that further addition of RNA beyond 1:0.5 stoichiometry of 3C/RNA resulted in the disappearance of peaks in a nonspecific manner (Figure S15), complicating analysis and preventing assessment of binding affinity by NMR.

### 3.2. AUC Reveals Multimeric Complex Formation Between PV-3C and RNA

**Figure 2:**
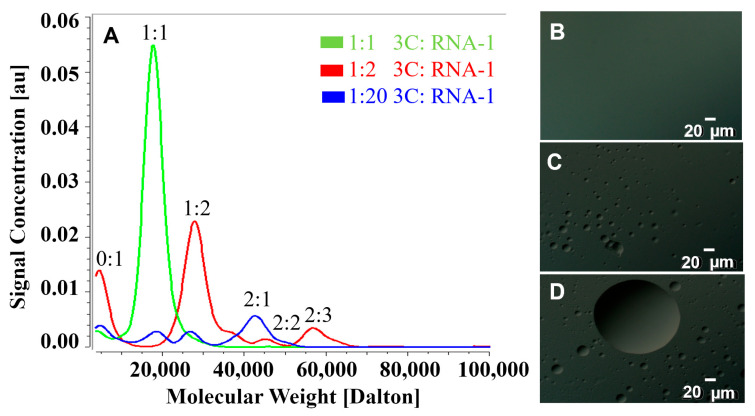
Interactions between PV-3C and RNA lead to multimeric complexes and liquid–liquid phase separation. (**A**) AUC of PV-3C with RNA-1, with PV-3C and RNA-1 forming a complex (green) at 1:1 molar stoichiometry. Upon increasing the RNA concentration (1:2 3C/RNA-1, red; 1:20 3C/RNA-1, blue), various multimolar ratio complexes appear as indicated in red and blue, respectively. (**B**) Using DIC microscopy, LLPS is not apparent with 1:1 3C/RNA-1 complex. (**C**,**D**) However, at higher ratios (panel (**C**), 1:2 3C/RNA-1; panel (**D**), 1:20 3C/RNA-1), DIC microscopy affirms the formation of LLPS. The PV-3C protein was at a concentration of 25 µM. RNA was added according to protein stoichiometry. The objective magnification was 40×. Both protein and RNA were in a buffer containing 10 mM HEPES and 50 mM NaCl, at pH 7.5. Experiments were conducted at 298 K.

NMR experiments with higher concentrations of RNA led to an overall decrease in peak intensities (Figure S15), which might be explained by the formation of multimeric complexes with 3C and RNA. To test this possibility, we utilized Sedimentation Velocity Analytical Ultracentrifugation (SV-AUC), which provides information about the oligomerization of macromolecules in solutions. In the absence of RNA, PV-3C is almost entirely monomeric (Figure S16). At a 1:1 molar ratio of PV-3C and RNA-1, there is evidence of only a single species (Figure 2 and Figure S17). However, at higher ratios of RNA (i.e., 1:2 3C/RNA-1 and above), various multimeric complexes appear, likely owing to oligomerization (Figure 2 and Figure S17). At the 1:2 3C/RNA-1 ratio, the major complex seems to be one that contains one 3C and two RNA, which would be consistent with the two potential RNA interaction sites on 3C (Figure 1).

To confirm whether multimeric complex formation was a general phenomenon, we also tested RNA-2 (5′-CAUACUGUUGUAGGGGAA-3′, derived from oriR, Table S1) binding with 3C through NMR and AUC (Figures S18 and S19, respectively). At 1:0.5 molar ratio of 3C/RNA, most of the peaks are absent from the SOFAST-HMQC spectra, which continues further to the complete absence of peaks at 1:1 molar ratio of 3C/RNA (Figure S18). These results may suggest a stronger interaction between 3C and RNA-2 and/or a higher propensity to form multimeric complexes. Indeed, multimeric complexes involving 3C and RNA-2 appear even at a 1:1 molar ratio (Figure S19).

### 3.3. Condensate Formation Observed from Differential Interference Contrast Microscopy

While AUC can detect changes in sedimentation behavior that suggest multimolar ratio complex formation and/or phase separation, it does not provide direct visual confirmation. Differential Interference Contrast (DIC) microscopy has been widely employed to study liquid–liquid phase separation (LLPS) [32,33]. Here, DIC microscopy suggested the formation of microscopic phase-separated droplets owing to RNA addition to 3C, but only under conditions that lead to multimeric complex formation. For instance, the 1:1 3C/RNA-1 complex does not show evidence of phase separation (Figure 2B), unlike those samples using 1:2 and 1:20 3C/RNA-1 ratios (Figure 2C,D, respectively). Further addition of protein or RNA does not affect the process of LLPS. PV-3C or RNA by itself does not lead to the same behavior; notably, 3C is predominantly in its monomeric form up to a 50 µM concentration (Figure S16).

In contrast, even a 1:1 mixture of PV-3C and RNA-2 shows evidence of LLPS (Figure S19), and several higher-order multimeric complexes observed by AUC for this sample (Figure S19). This finding suggests that RNA-2 is more capable of seeding oligomerization of PV-3C, leading to LLPS. As LLPS formation tends to be salt-dependent, we also investigated the effect of varying NaCl concentrations on LLPS of 3C with RNA-1 and RNA-2. We observed that LLPS persisted up to 400 mM NaCl. At 500 mM NaCl, both samples showed complete deformation of condensates, leading to the formation of aggregates (Figure S20). LLPS was also apparent for all other RNAs tested (Figure S21), suggesting this is a general phenomenon for 3C interactions with RNA oligonucleotides.

### 3.4. PV-3CD and RNA Interactions Also Lead to LLPS

**Figure 3:**
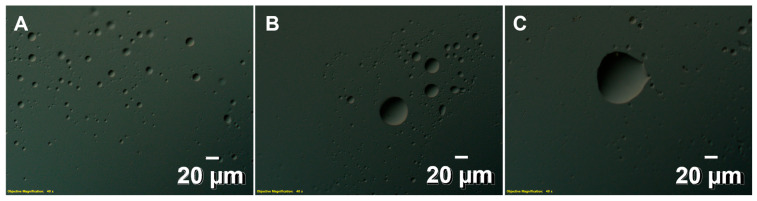
Interactions between PV-3CD and RNA lead to LLPS. DIC microscopy shows evidence for LLPS under all 3CD to RNA ratios tested (panel (**A**), 1:0.5; panel (**B**), 1:1; panel (**C**), 1:2). The PV-3CD protein was at a concentration of 25 µM. RNA was added according to protein stoichiometry. The PV-3C protein was at a concentration of 25 µM. The objective magnification was 40×. Both protein and RNA were in a buffer containing 10 mM HEPES, 50 mM NaCl, and at pH 7.5. Experiments were conducted at 298 K.

Having examined the effect of RNA on PV-3C, we also confirmed that addition of RNA-1 to PV-3CD likewise led to LLPS, as observed by DIC microscopy (Figure 3), under all conditions tested, including 1:0.5 and 1:1 mixture of RNA and protein. Similarly, all RNAs tested with 3CD led to LLPS according to DIC microscopy (Figure S22). These findings were consistent with the observation of larger oligomers in the 20–40 nm range according to negative-stain electron microscopy (Figure S23).

### 3.5. Molecular Model of the Multivalent Interactions Between PV-3C/3CD and RNA

**Figure 4:**
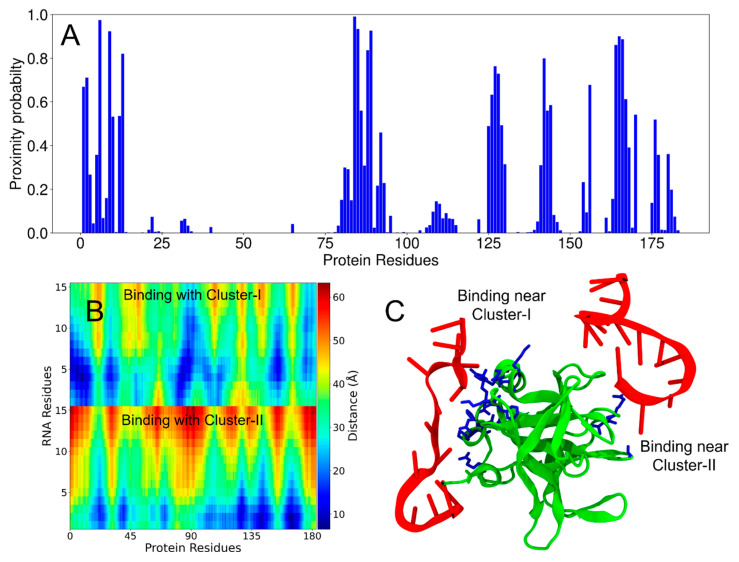
Two interaction sites between PV-3C protein and RNA-1 by MD simulations. (**A**) Proximity probabilities (Pprox) of residues in 3C. (**B**) Distance map between the residues of RNA-1 and 3C. The color bar represents the color-coded distances underlying the distance map. The vertical axis shows the RNA residues of the two chains (each with 15 nucleotides). (**C**) Simulation snapshot showing two chains of RNA-1 interacting with the residues in Cluster-I and Cluster-II of the 3C protein (as also observed from NMR experiments). The protein and RNA chains are shown in green and red, respectively. The residues colored blue have proximity probabilities greater than 0.5.

To gain more insight into the multivalent nature of protein–RNA interactions, which are likely critical for LLPS, we performed molecular dynamics (MD) simulations of the PV-3C and PV-3CD proteins interacting with RNA-1 and RNA-2 [41,42,43]. In our analysis, RNA was considered bound to the protein when any RNA heavy atom (non-hydrogen atom) was located within 5 Å of any protein residue. Using this geometric criterion, we defined a proximity probability (Pprox) for each protein residue as Pprox = n/N, where n represents the number of timeframes during which any heavy atom of the given residue is bound to RNA, and N is the total number of frames in the MD trajectory. Thus, Pprox effectively quantifies the residence time of RNA molecules near protein binding sites throughout the simulation (Figure 4A and Figure S24A).

MD simulations encompass two primary types of non-bonded interactions: electrostatic (Coulomb potential) and van der Waals (Lennard-Jones potential). Protein–nucleotide interactions predominantly arise from electrostatic forces, driven by the negatively charged nucleotides interacting with positively charged protein regions (Figure 1C) [43]. However, in this study, we exclusively used the geometric criterion described above to determine protein–RNA interactions and did not explicitly account for energetic contributions (because energy values are force-field dependent). Consequently, a high Pprox value indicates protein regions frequently in proximity to RNA segments during the simulation. Such regions can include residues adjacent to the actual interaction sites, some of which may not exhibit significant chemical shift perturbations in NMR experiments. Therefore, direct one-to-one comparisons of interacting residues between computational and experimental results should be approached cautiously.

In the 3C simulations, both RNA-1 and RNA-2 exhibited substantial interactions with Cluster-I residues (Figure 4 and Figure S24). Both RNA oligonucleotides also interacted with Cluster-II residues, although the binding propensity for Cluster-II was apparently lower than that for Cluster-I (Figure 4A and Figure S24A). RNA-2 also displayed a higher binding propensity compared to RNA-1 for both residue clusters (Figure S24). Distance maps between RNA molecules and 3C also summarize the average interactions throughout the trajectory (Figure 4B and Figure S24B; blue indicating shorter distances between protein and the two RNA molecules). The distance maps are intended to show the entire range of sampled distances over the course of the simulation. MD simulation videos provide a clear visual representation of the 3C and RNA interaction (Videos S1 and S2).

**Figure 5:**
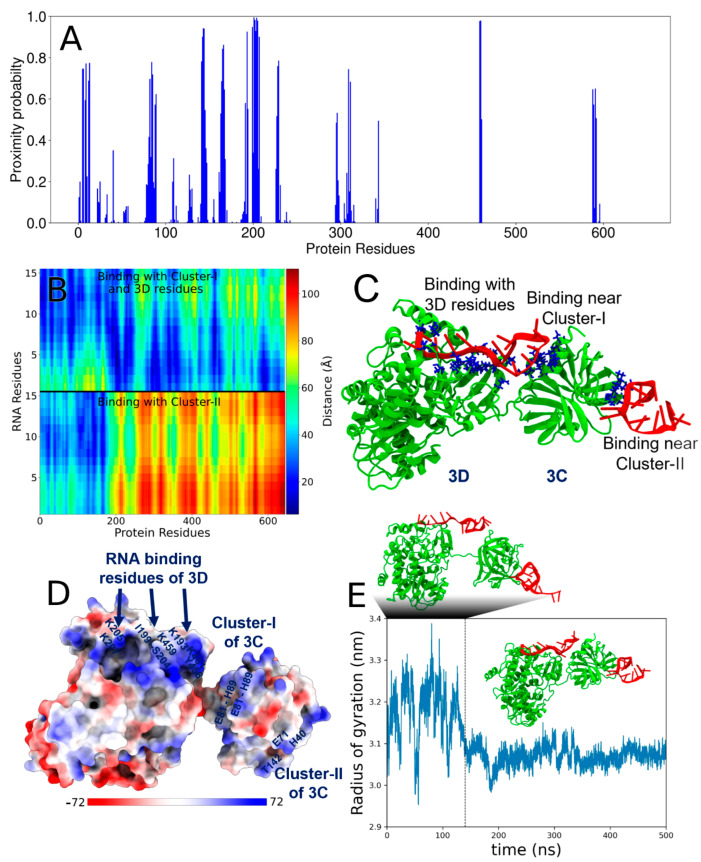
Interactions between PV-3CD protein and RNA-1 from MD simulations. (**A**) Proximity probabilities (Pprox) of residues in 3CD. (**B**) Distance map between the residues of RNA-1 and 3CD. The color bar represents the color-coded distances underlying the distance map. The vertical axis shows the RNA residues of the two chains (each with 15 nucleotides). (**C**) Simulation snapshot showing two chains of RNA-1 interacting with the residues in Cluster-I and Cluster-II of the 3CD protein. The former also interacts with positively charged residues in the 3D domain. The protein and RNA chains are shown in green and red, respectively. The residues colored blue have proximity probabilities greater than 0.5. (**D**) Electrostatic map of the PV-3CD protein surface. (**E**) Time evolution of the radius of gyration (Rg) of PV-3CD obtained from MD simulations. Representative snapshots of the protein structures in these two distinct states are presented along with the Rg time trace (green represents protein and red represents RNA with the dashed line representing a transition between the two distinct states.).

Given that the MD simulations were largely consistent with the PV-3C NMR results, we extended this methodology to investigate RNA binding to full-length PV-3CD. While we have previously published NMR results with PV-3CD [4], these studies were limited to Ile δ1-^13^CH_3_ resonances, which would provide limited information about RNA binding. Similar to the results with PV-3C, residues in Cluster-I and Cluster-II showed propensity to interact with RNA (Figure 5 and Figure S25). Additionally, and perhaps not surprisingly given that 3D is the RNA-dependent RNA polymerase, RNA also interacted with the 3D domain of PV-3CD.

Like 3C, the binding propensity for Cluster-II in 3CD was apparently lower than that for Cluster-I with both RNA oligonucleotides (Figure 5A and Figure S25A). Notably, there is a contiguous surface of positively charged groups connecting 3C and 3D domains (Figure 5D), likely helping to facilitate RNA binding due to the proximity of RNA-binding regions at the interface between these domains. Intriguingly, RNA binding also apparently induces proximity and compaction between the 3C and 3D units within the PV-3CD protein, as shown by a distinct structural transition, characterized by a significant decrease in Rg, observed around 130 ns of the simulation (Figure 5E). 3CD-RNA-1 forms a stable complex as concluded from Rg (Figure 5E), because the stable complex exhibits a relatively constant Rg over the simulation time after binding, while fluctuations in Rg indicate dynamic regions or transient interactions within the complex. MD simulation videos provide a clear visual representation of the 3CD and RNA interaction (Videos S3 and S4).

## 4. Discussion

Besides their proteolytic function, 3C and/or 3CD engage with RNA control elements in the picornavirus genome to help regulate and coordinate transcription and translation events [1,2,3,4]. Previous studies have brought insight into how PV-3C interacts with oriI, but little to no information has been available for similar interactions with oriL- and oriR-derived RNA [14]. It is especially notable that proposed RNA sites for 3C/3CD interaction have little to no sequence and/or structure similarities. As such, we investigated the structure and sequence specificity of RNA oligonucleotide binding to PV 3C and 3CD. While our studies revealed little specificity underlying 3C–RNA or 3CD–RNA interactions (at least using this set of RNA oligonucleotides), both sets of interactions can induce LLPS, a phenomenon that is important to various viral processes.

It is now well-established that LLPS is a complex process triggered by multivalent interactions, weak forces, and the interplay between proteins and RNA [19,20,48,49,50]. For 3C, NMR identified two potential regions of RNA interaction: Cluster-I, including amino acid residues in the 80’s region (as identified in previous NMR studies) and the N-terminal alpha helix, and Cluster-II on the opposite face around/adjacent to the protease active site (Figure 1) [4]. Similar regions in Coxsackievirus 3C as those in Cluster-I of PV-3C have also been determined to form interactions with the corresponding cloverleaf stem-loop D RNA. Interestingly, most of the identified residues belong to loops of PV-3C; the inherent flexibility in these loops may allow the residues to explore different conformations to allow for interactions with a variety of RNA sequences. Notably, both Cluster regions have positively charged surface potentials through which electrostatic interactions with negatively charged RNA could occur (Figure 1C). Results from MD simulations (Figure 4 and Figure S24) are also consistent with the two RNA-binding sites. Results from SV-AUC for the 3C/RNA-1 complex at a 1:2 ratio are likewise consistent with two RNA interactions for one 3C protein (Figure 2).

The connection between multivalency and LLPS formation is especially shown with the interactions involving 3C and RNA-1. With an equimolar mixture, only a 1:1 protein–RNA complex is observed by SV-AUC (Figure 2A). Under these conditions, there is no apparent LLPS by DIC microscopy (Figure 2B). However, as the RNA concentration increases, multimeric complexes form (Figure 2A), which likely lead to LLPS (Figure 2C,D). Multimeric complexes using RNA-2 are apparent at the lower protein–RNA ratio (Figure S19), and so is LLPS (Figure S19). The computational results also suggest that RNA-2 has a stronger ability to interact with Cluster-I and especially with Cluster-II residues (Figure S24), providing a molecular basis for its increased propensity for multimeric complex formation and LLPS formation. Our findings also demonstrate that LLPS formation occurs across a broad range of salt concentrations, maintaining LLPS up to 400 mM NaCl (Figure S20). However, beyond this threshold, high ionic strength (500 mM NaCl) drives a distinct phase transition to amorphous aggregates, highlighting the critical role of electrostatic interactions in LLPS formation through protein–RNA interactions.

**Figure 6:**
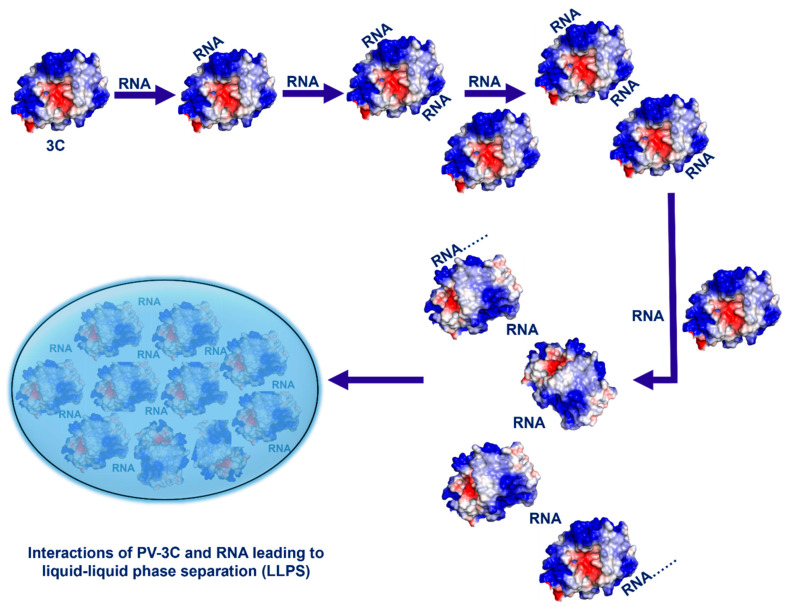
Multivalent interactions between 3C (or 3CD) and RNA oligonucleotides lead to liquid–liquid phase separation.

The phenomena observed for RNA-1 and RNA-2 appear to be general, as all other 3C–RNA interactions lead to decreases in NMR peak intensities (consistent with the formation of large multimeric complexes; Figures S2–S14), and there is evidence for LLPS by DIC microscopy (Figure S21). Moreover, this phenomenon likely extends to other picornaviral 3C proteins given their conservation of sequence (Figure S26), structure (Figure S27), and electrostatic surface potential (Figure S28). Comparative sequence and electrostatic analyses across representative picornaviral 3C proteases, including PV-3C, CVB-3C, EV71-3C, FMDV-3C, and HAV-3C, demonstrate strong conservation of the KFRDI/KFRGI motif and adjacent basic residues in Cluster-I, as well as preservation of the positively charged Cluster-II surface near the catalytic site (Figures S26–S28). This conservation suggests that multivalent RNA interactions and LLPS formation are likely to represent conserved mechanistic feature of 3C proteases across the Picornaviridae family. Altogether, our results suggest a mechanism by which interactions between RNA oligonucleotides and 3C induce LLPS (Figure 6).

It is noteworthy that Cluster-II residues, which exhibit RNA-induced chemical shirt perturbations, are positioned adjacent to the catalytic triad (H40, E71, C147), suggesting that RNA can transiently engage the catalytic surface. Although the C147A variant used here precludes direct assessment of catalytic effects, this spatial overlap implies potential regulatory coupling between RNA binding and protease function. RNA association at Cluster-II could modulate active site accessibility, influence local conformational dynamics, or help spatially position 3C/3CD within replication complexes. Similar allosteric coupling between catalytic and RNA-binding regions has been proposed for other enteroviral 3C proteases [51]. This supports the possibility that RNA-mediated modulation of protease activity represents an additional regulatory mechanism during viral replication.

We should note a caveat to the current studies regarding RNA specificity. While the 15-nt RNA oligonucleotides used in this study were derived from regions within the oriL and oriR elements, they do not encompass the full cis-acting replication elements. Therefore, our observation of limited sequence or structural specificity likely reflects the use of truncated RNA fragments that capture local binding motifs but not the full secondary or tertiary architecture of the native elements. Indeed, recent work by Dias-Solange et al. [51] demonstrated that longer RNAs exhibit enhanced specificity and cooperative interactions with viral replication proteins. Together, these findings suggest that 3C and 3CD possess an intrinsic ability to interact broadly with RNA, while higher-order specificity and regulatory effects may emerge in the context of full-length replication elements or assembled replication complexes.

Given the results with 3C, it is not surprising that RNA interactions with 3CD also lead to LLPS (Figure 3 and Figure S22), as the 3D domain also interacts with RNA. MD simulations also suggest that RNA has propensity to interact with the corresponding Cluster-I and Cluster-II residues, and moreover, the Cluster-I RNA-binding surface appears to be contiguous with an RNA-binding surface on the 3D domain (Figure 5). Given that 3CD is more abundant in cells than 3C, these results may be more biologically relevant [6,52,53,54,55]. In either case, protein–RNA-induced LLPS seems to be largely independent of the RNA sequence, such that 3C and/or 3CD could form such interactions with any RNA in the cell, not only those involving the viral RNA replication elements. These findings suggest that there may be mechanisms to prevent and/or leverage 3C/3CD-involved LLPS.

The 3C/3CD concentrations used in our in vitro studies (~25 µM) were chosen to ensure robust detection in NMR, DIC microscopy, and other biophysical assays. While likely higher than average cytosolic levels of 3C or 3CD, these concentrations are within the range commonly used for in vitro LLPS studies and are consistent with transient micromolar enrichment observed for viral replication proteins, including poliovirus 3D, enterovirus 71 3C/3D, and SARS-CoV-2 N, within replication organelles or stress-granule-like compartments [19,48,49,50,56,57,58,59]. Other viral and host proteins, such as measles virus N–P complexes and enterovirus 3D polymerase, similarly undergo LLPS at comparable concentrations. Thus, the LLPS observed here likely reflects a biologically relevant phenomenon that may occur in regions of high local 3C/3CD concentration during infection.

LLPS is increasingly recognized as a key component in driving the viral life cycle, influencing viral replication, assembly, and host–pathogen interactions [56,57,58,59]. For example, LLPS may be involved in the formation of viral replication compartments, which may help in evading host cell immune surveillance and virus assembly, and interfere with host stress response [56,57,58,59]. For the latter, stress granules (SGs) and processing bodies (PBs) are both cytoplasmic ribonucleoprotein complexes that interact dynamically, exchanging mRNA and proteins to regulate translation, storage, and degradation of mRNA under stress conditions, including virus infection [60,61,62]. It is known that 3C expression inhibits SG formation and leads to PB dispersal, likely through its ability to cleave key protein factors G3BP1, important for nucleating SGs, and Dcp1a and PAN3, key components of PBs [60,61,62]. In contrast, the expression of PV-3CD has no apparent effect on SG assembly, although it has a similar effect on PB dispersal as PV-3C [61]. PV-3CD is also known to interact with many components of SGs, including eIF4G, GTPases, G3BP1, TIA1, PCBP, and PABP [63,64,65,66,67,68,69,70]. It is also a key factor in the generation of virus replication organelles, although the relationship between these membrane-bound structures and the membraneless SGs and PBs is unclear [71,72,73,74]. As such, our results suggest further ways that 3C and/or 3CD might interact with protein–RNA condensates, potentially by first integrating into these complexes before cleaving essential proteins to cause disruption. LLPS, stimulated by interactions between RNA and 3C/3CD, could also promote the disassembly of SGs and PBs. Thus, the mechanisms revealed in this study hint towards possible functional and regulatory roles for 3C and/or 3CD in virally induced LLPS-related events, including in stress responses.

## Data Availability

All data supporting the findings of this study are available within the article and Supplementary Materials. Additional raw data and materials are available from the corresponding author upon reasonable request. Where applicable, datasets have been deposited in publicly accessible repositories and accession numbers are provided in the manuscript.

## Supplementary Materials

The following supporting information can be downloaded at: https://www.mdpi.com/article/10.3390/v17111473/s1, Table S1: List of RNA sequences utilised for experiments with the origin; Figure S1: Predicted secondary structure of oriL and oriR from the PV RNA genome; Figure S2: PV-3C and RNA-3 (UUUUUUUUUUUUUUU) binding; Figure S3: PV-3C and RNA-4 (ACCCCAGAGGCCCAC) binding; Figure S4: PV-3C and RNA-5 (CCAUCAAGAAUCCUA) binding; Figure S5: PV-3C and RNA-6 (AAAAAAAAAAAAAAA) binding; Figure S6: PV-3C and RNA-7 (CGCAGCAAACACAAG) binding; Figure S7: PV-3C and RNA-8 (CUACCUCAGUCGAAU) binding; Figure S8: PV-3C and RNA-9 (CGUUGGCUUGACUCA) binding; Figure S9: PV-3C and RNA-10 (GGGGUUGUACCCACCCC) binding; Figure S10: PV-3C and RNA-11 (CUCCGGUAUUGCGGUACCCUUG) binding; Figure S11: PV-3C and RNA-12 (GAUUGGGUCAUACUGUUGUAG) binding; Figure S12: PV-3C and RNA-13 (CAGAGGAACACGUGGCGGCG) binding; Figure S13: PV-3C and RNA-14 (UUUUCUUUAAUUCGGAGAAAA) binding; Figure S14: PV-3C and RNA-15 (AGUUCAAGAGC) binding; Figure S15: Disappearance of NMR peaks at higher ratios of 3C: RNA-1 suggests multimeric complex formation; Figure S16: SV-AUC of PV-3C only; Figure S17: SV-AUC of PV-3C; Figure S18: Titration with RNA-2 leads to disappearance of NMR peaks at even lower 3C to RNA ratios; Figure S19: Multimeric complex formation between PV-3C and RNA-2 leads to LLPS formation; Figure S20: Salt effect on LLPS formation of 3C with RNA-1 and RNA-2; Figure S21: LLPS formation of PV-3C with RNA-3–RNA-15 observed through DIC microscopy; Figure S22: LLPS formation of PV-3CD with RNA-3–RNA-15 observed through DIC microscopy; Figure S23: EM analysis of 3CD-RNA-1 oligomerization; Figure S24: Interactions between PV-3C protein and RNA-2 from MD simulations; Figure S25: Interactions between PV-3CD protein and RNA-2 from MD simulations; Figure S26: CLUSTAL O (1.2.4) multiple sequence alignment of 3C proteases; Figure S27: The structural and sequential resemblance of PV-3C to other viral 3Cs; Figure S28: The electrostatic surface potential map for 3Cs; Video S1: Visualization of a molecular dynamics (MD) trajectory of the PV 3C:RNA-1 complex; Video S2: Visualization of a molecular dynamics (MD) trajectory of the PV 3C:RNA-2 complex; Video S3: Visualization of a molecular dynamics (MD) trajectory of the PV 3CD:RNA-1 complex; Video S4: Visualization of a molecular dynamics (MD) trajectory of the PV 3CD:RNA-2 complex.

## Abbreviations

The following abbreviations are used in this manuscript:

3C	3C main protease
3D	3D RNA-dependent RNA polymerase
CSP	Chemical shift perturbations
CREs	Cis-acting replication elements
HEPES	4-(2-hydroxyethyl)-1-piperazineethanesulfonic acid
HMQC	Heteronuclear multiple quantum coherence
HPLC	High-performance liquid chromatography
IPTG	Isopropyl β-D-1-thiogalactopyranoside
LLPS	Liquid–liquid phase separation
MD	Molecular dynamics
MEM	Minimum essential medium
MWCO	Molecular weight cutoff
NaCl	Sodium chloride
NH_4_Cl	Ammonium chloride
Ni-NTA	Nickel–nitrilotriacetic acid
NMR	Nuclear magnetic resonance
NpT	Constant number of particles, pressure, and temperature
NVT	Constant number of particles, volume, and temperature
OD600	Optical density at 600 nm
PDB	Protein Data Bank
PEI	Polyethyleneimine
PMSF	Phenylmethanesulfonyl fluoride
PV	Poliovirus
SARS-CoV	Severe acute respiratory syndrome-related coronavirus
SV-AUC	Sedimentation velocity analytical ultracentrifugation
TIA-1	T-cell intracellular antigen 1
VPg	Viral protein genome-linked
