# Supplementary figures and images for: Multivalent Interactions Between the Picornavirus 3C(D) Main Protease and RNA Oligonucleotides Induce Liquid–Liquid Phase Separation
Source: Viruses. 2025 Nov 4;17(11):1473. doi: 10.3390/v17111473 (PMC12656920; doi:10.3390/v17111473)

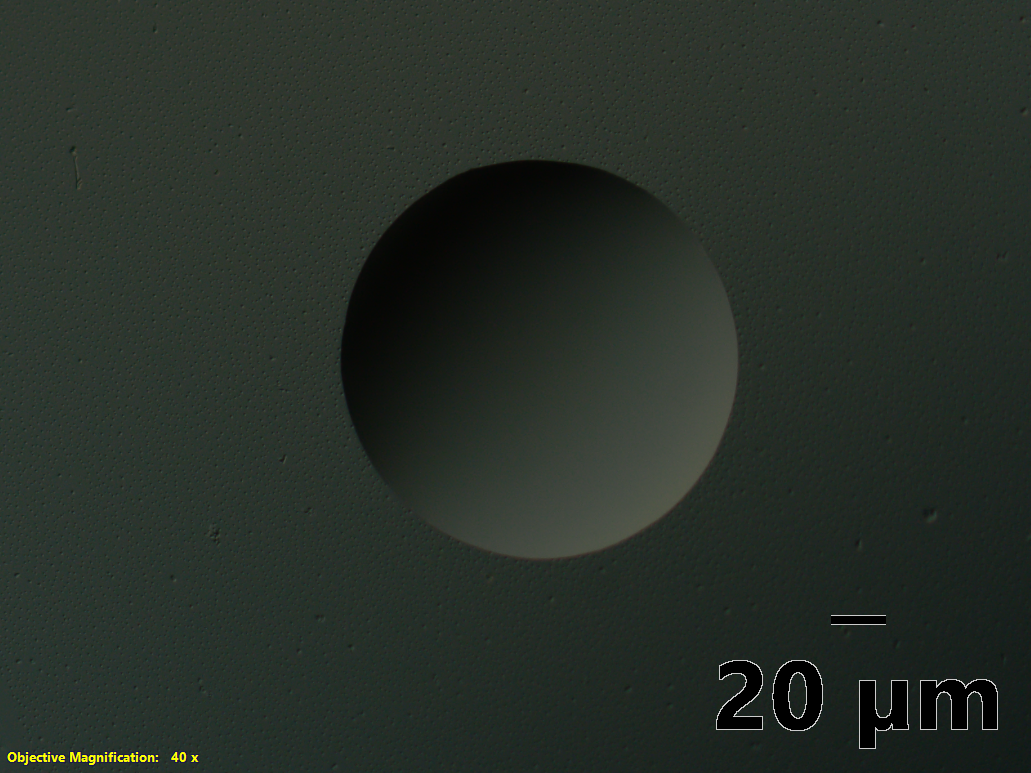

Supplement: Supplementary file 1 [file viruses-17-01473-s001.zip › Figure S19A.tif]

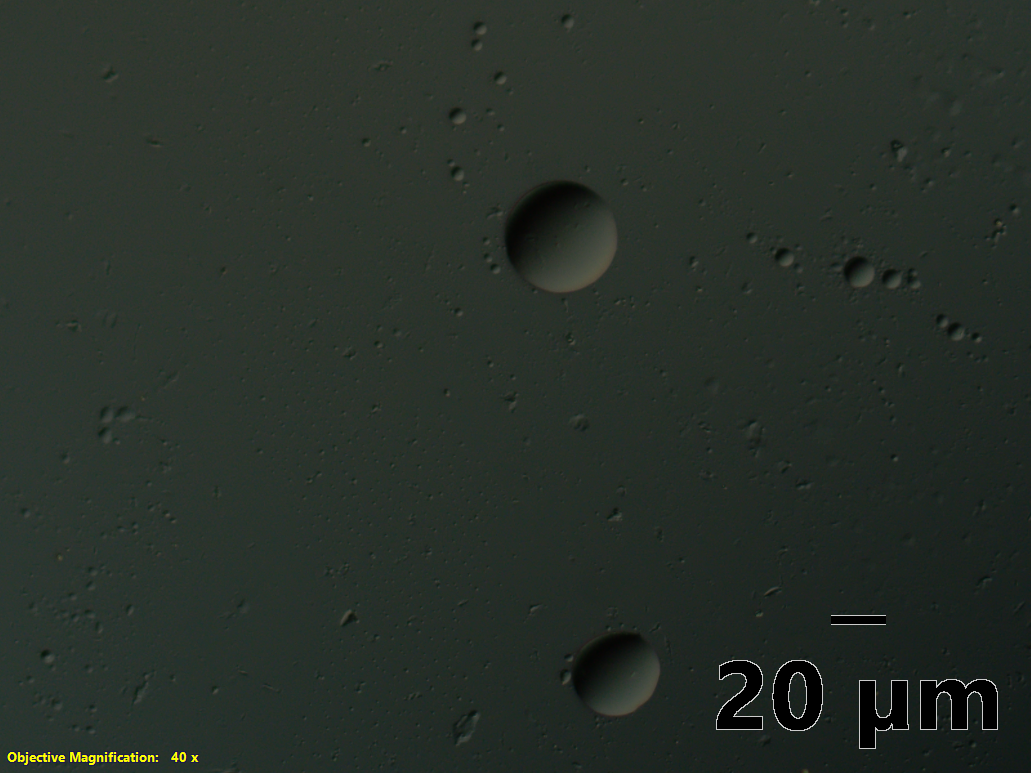

Supplement: Supplementary file 1 [file viruses-17-01473-s001.zip › Figure S21 3C RNA10.tif]

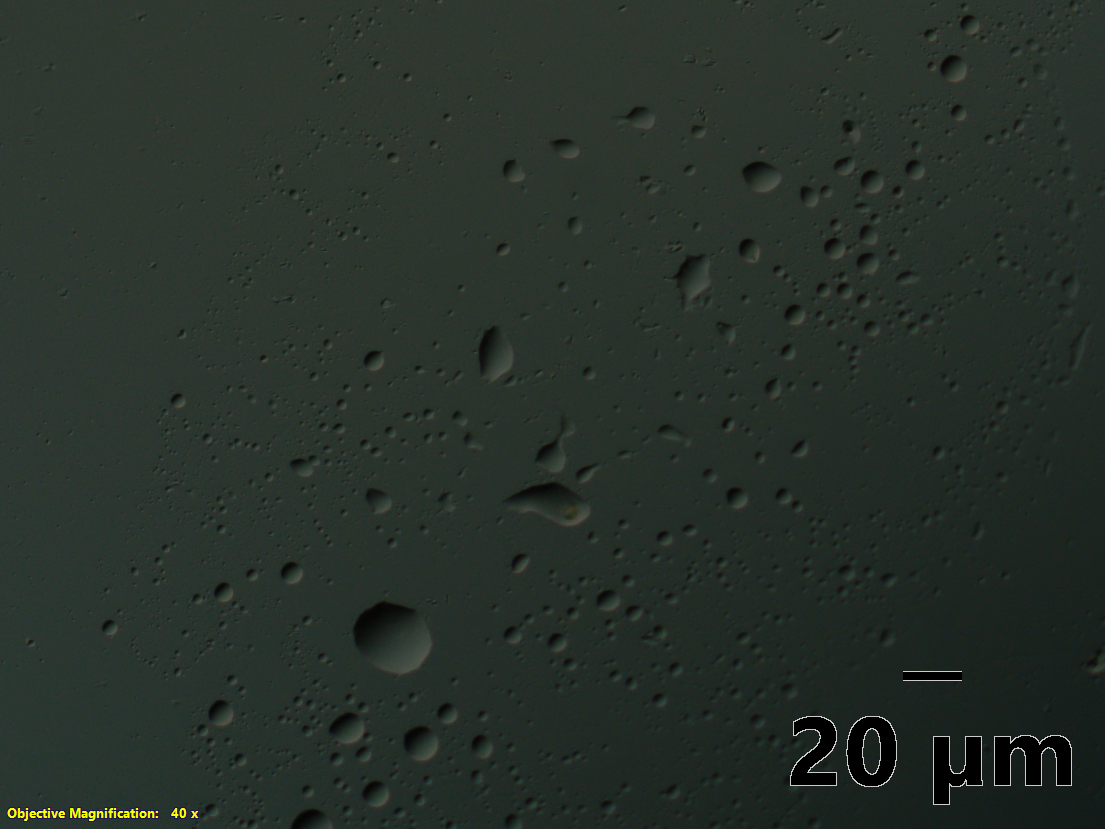

Supplement: Supplementary file 1 [file viruses-17-01473-s001.zip › Figure S21 3C RNA11.tif]

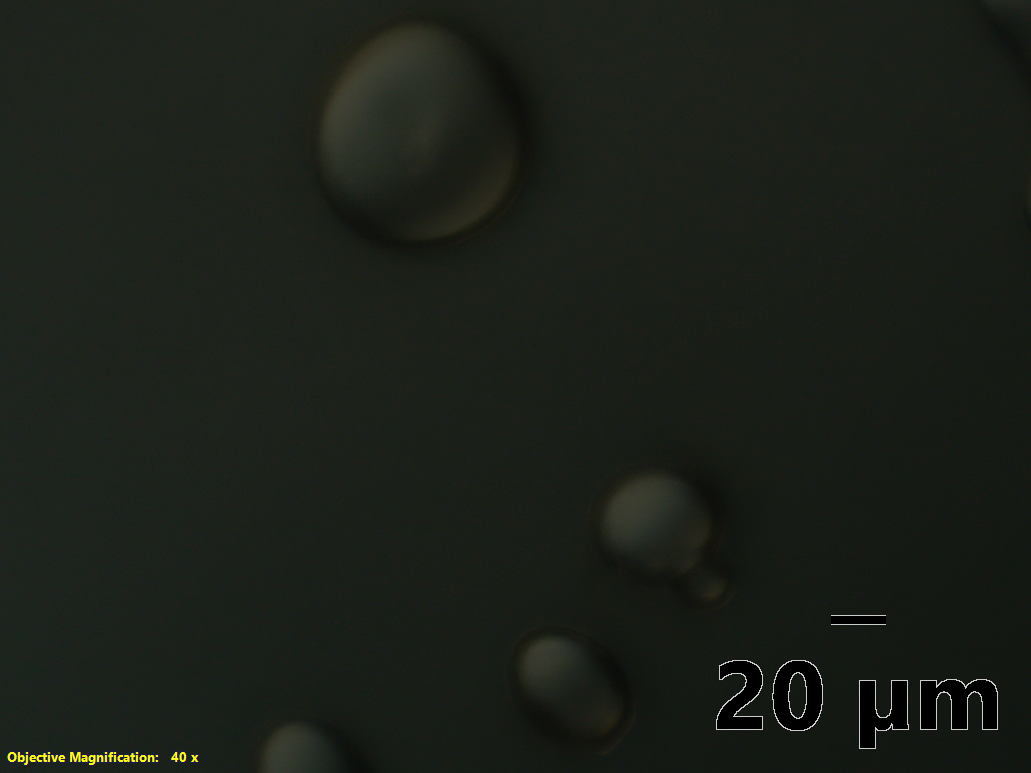

Supplement: Supplementary file 1 [file viruses-17-01473-s001.zip › Figure S21 3C RNA12.tif]

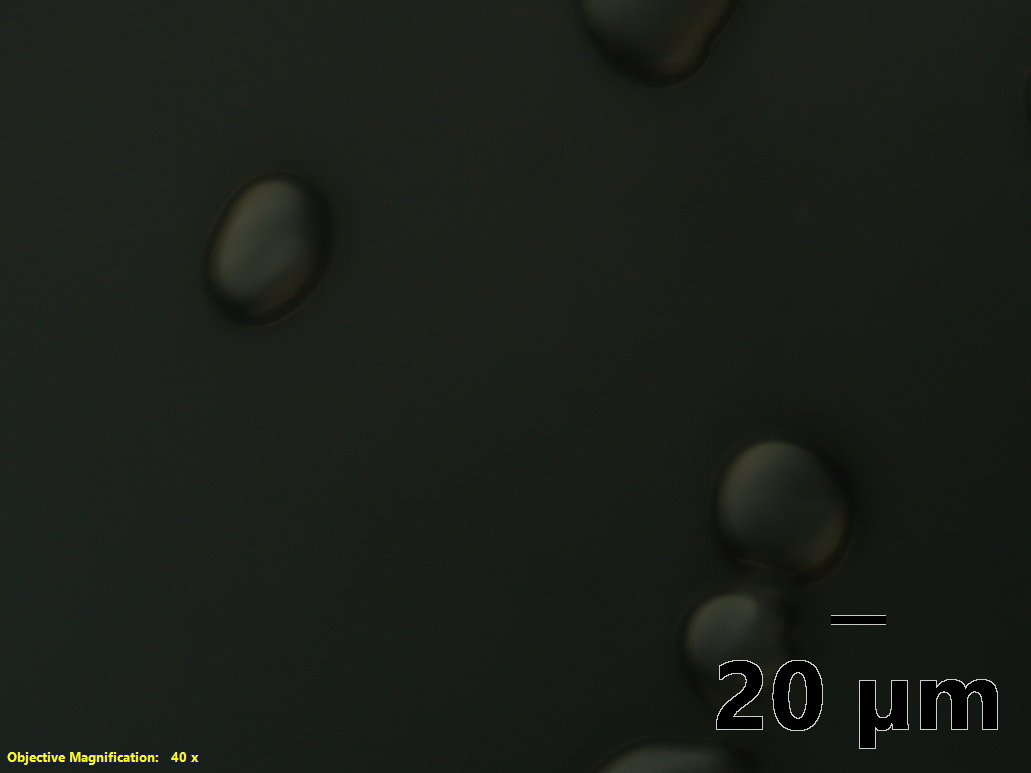

Supplement: Supplementary file 1 [file viruses-17-01473-s001.zip › Figure S21 3C RNA13.tif]

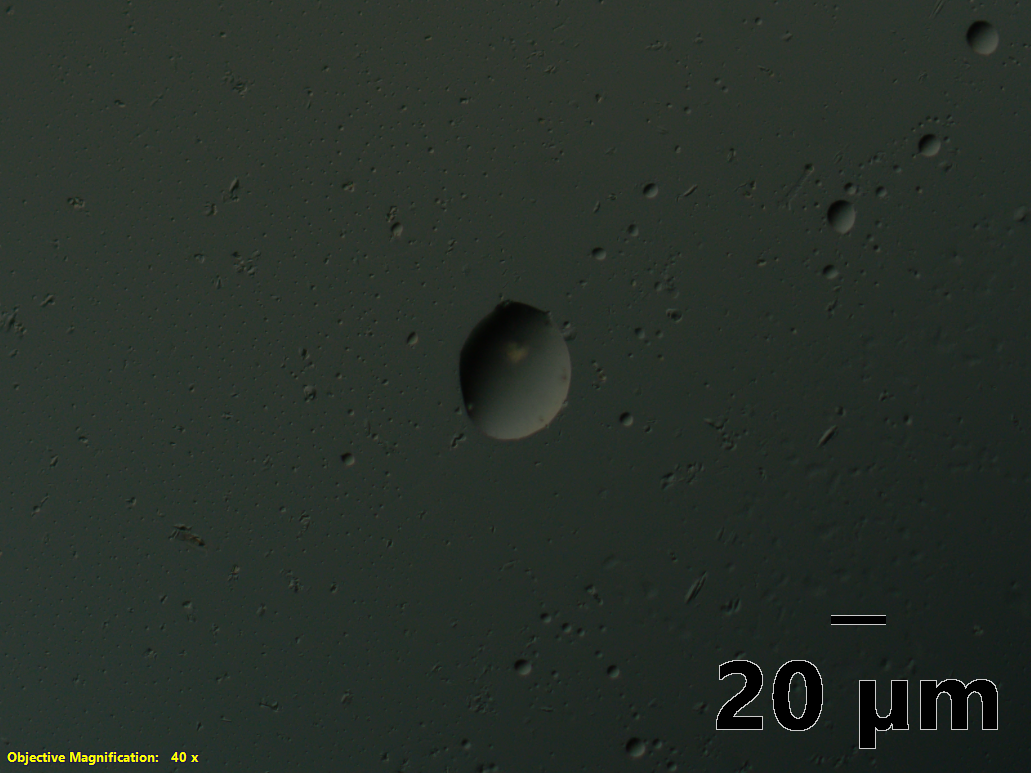

Supplement: Supplementary file 1 [file viruses-17-01473-s001.zip › Figure S21 3C RNA14.tif]

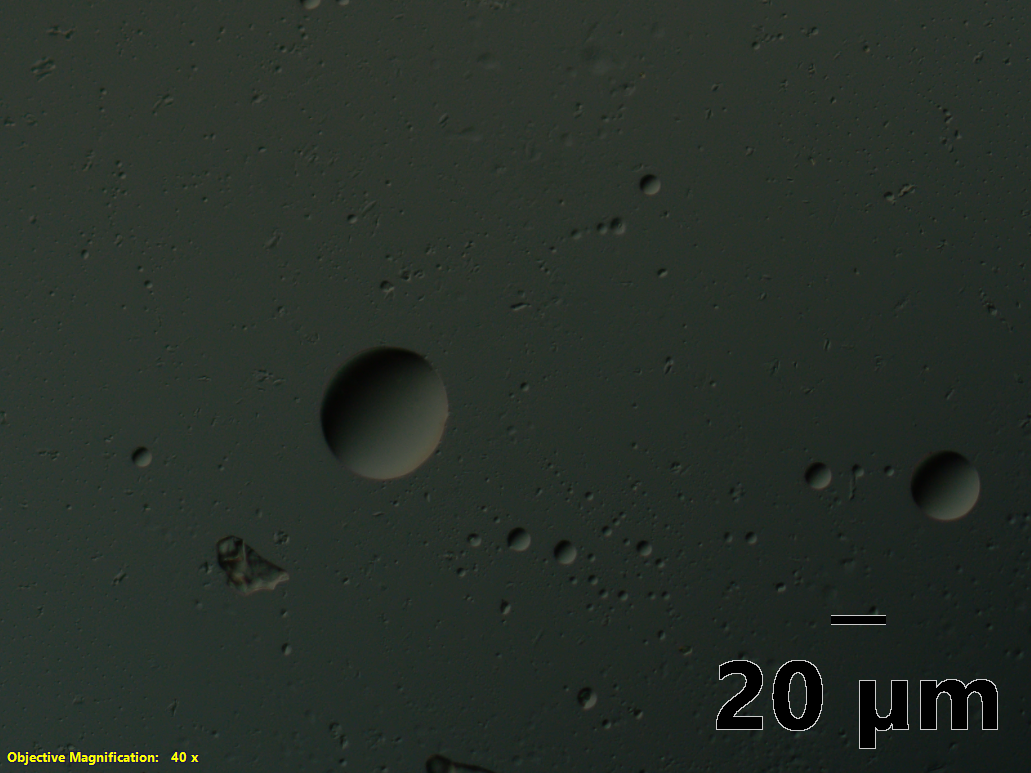

Supplement: Supplementary file 1 [file viruses-17-01473-s001.zip › Figure S21 3C RNA15.tif]

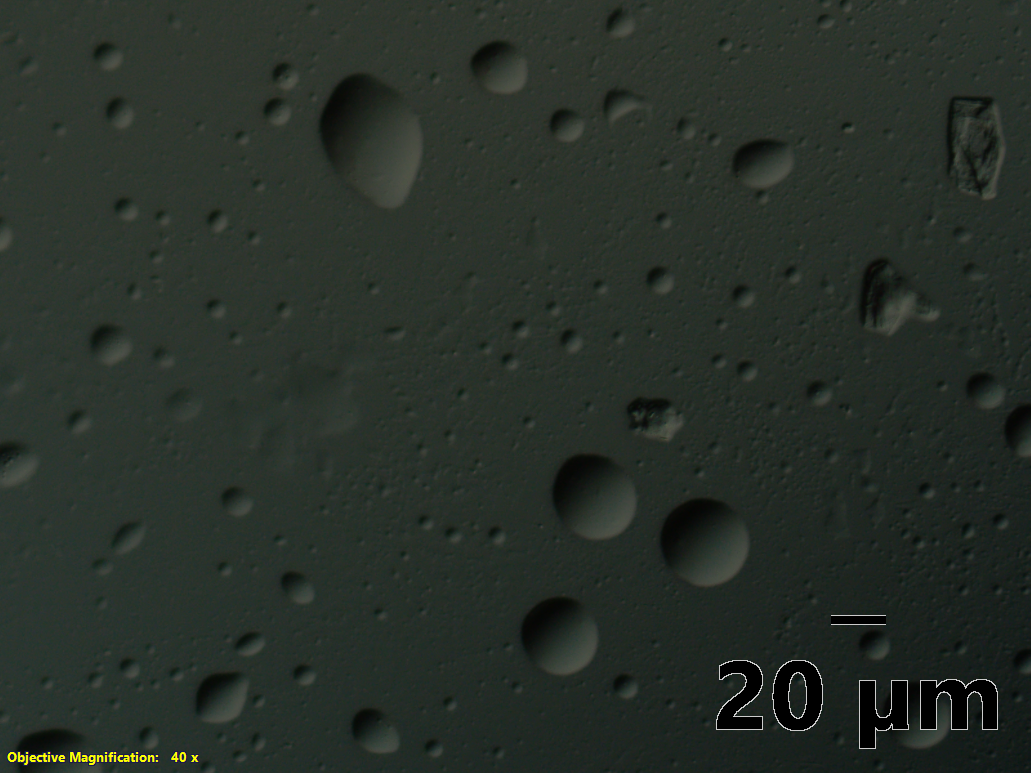

Supplement: Supplementary file 1 [file viruses-17-01473-s001.zip › Figure S21 3C RNA4.tif]

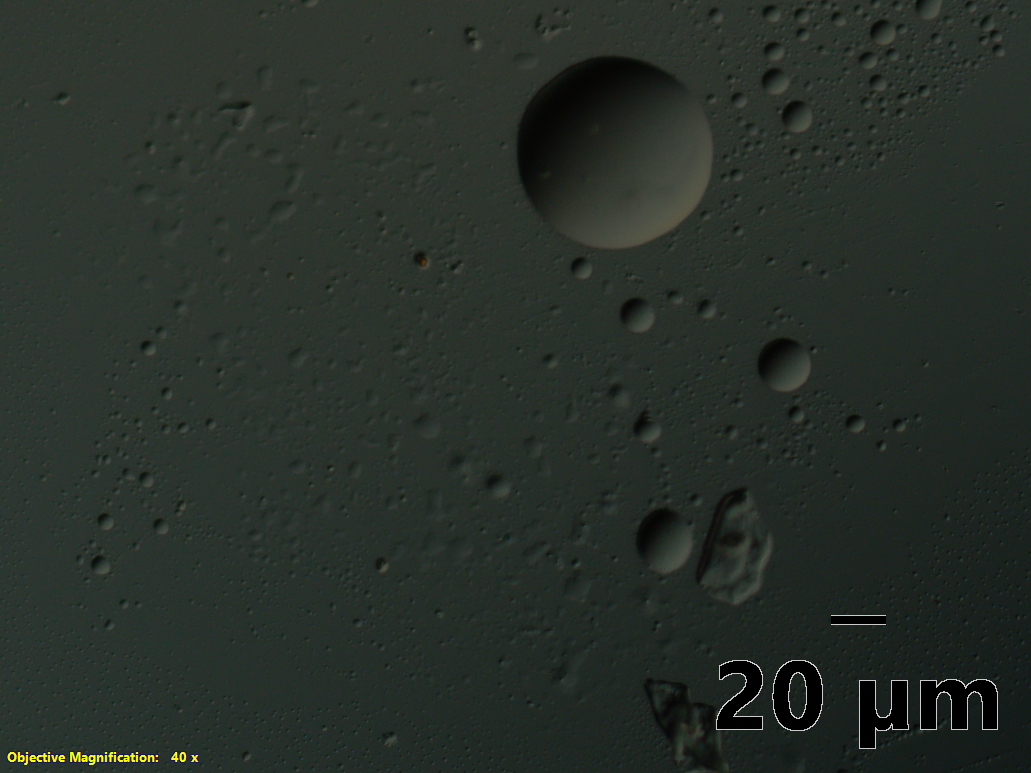

Supplement: Supplementary file 1 [file viruses-17-01473-s001.zip › Figure S21 3C RNA6.tif]

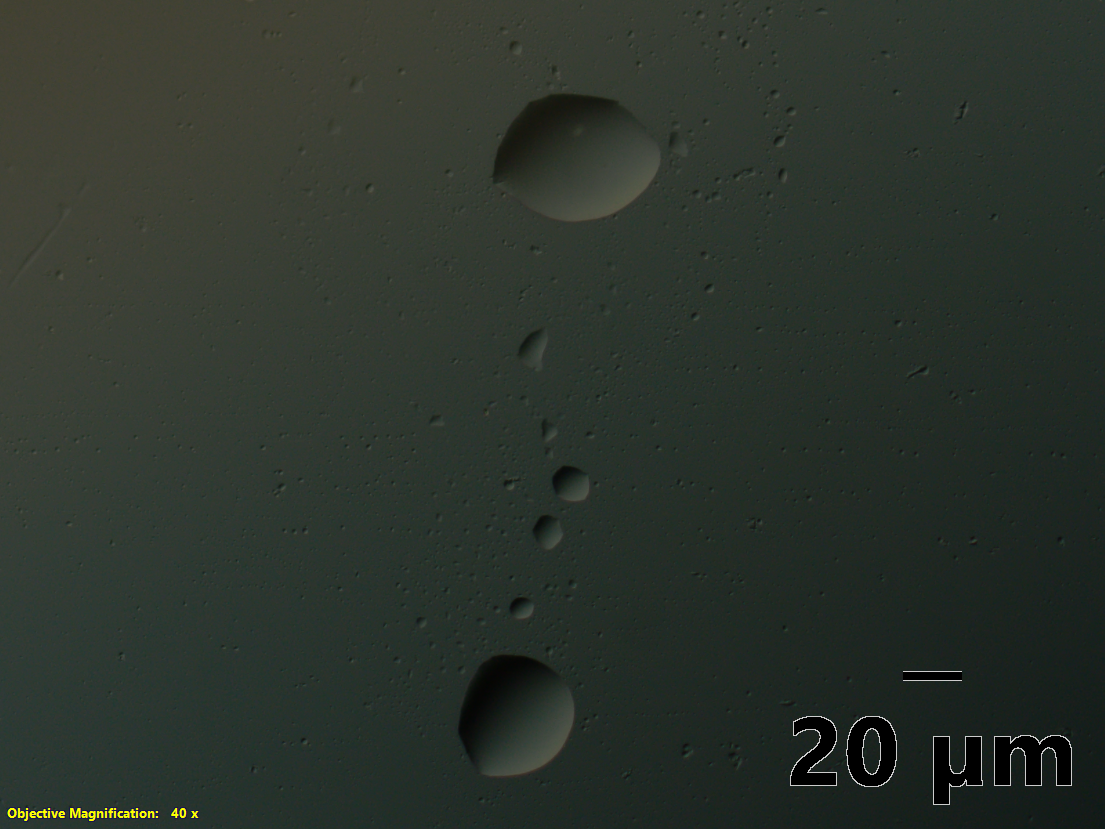

Supplement: Supplementary file 1 [file viruses-17-01473-s001.zip › Figure S21 3C RNA7.tif]

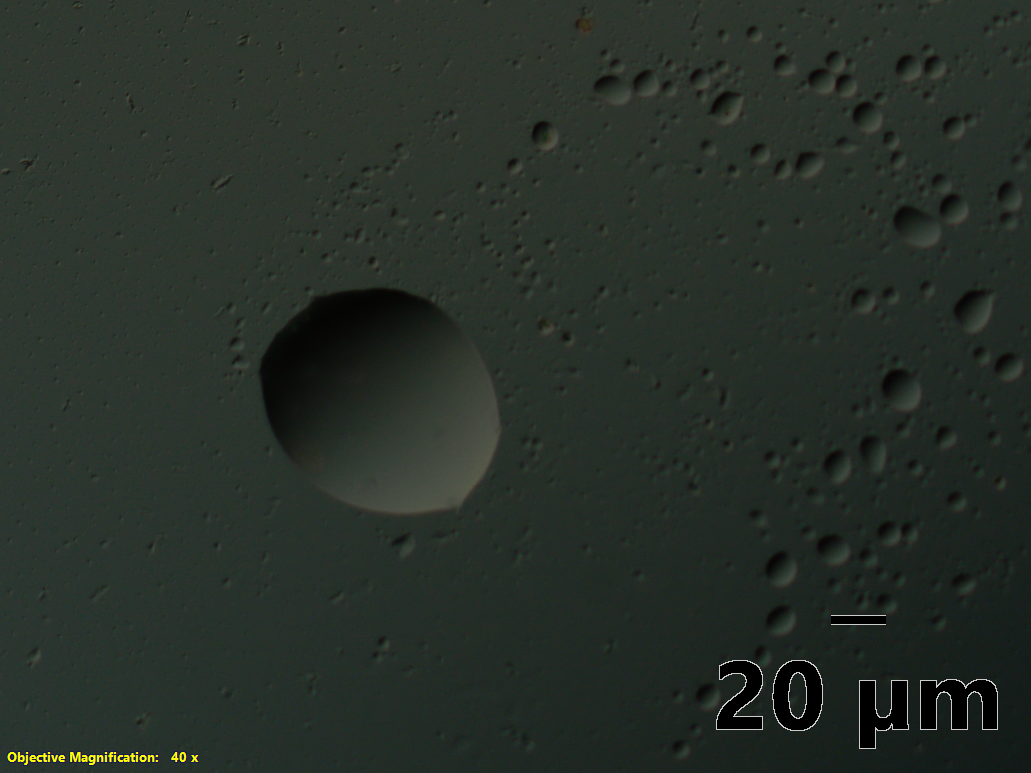

Supplement: Supplementary file 1 [file viruses-17-01473-s001.zip › Figure S21 3C RNA8.tif]

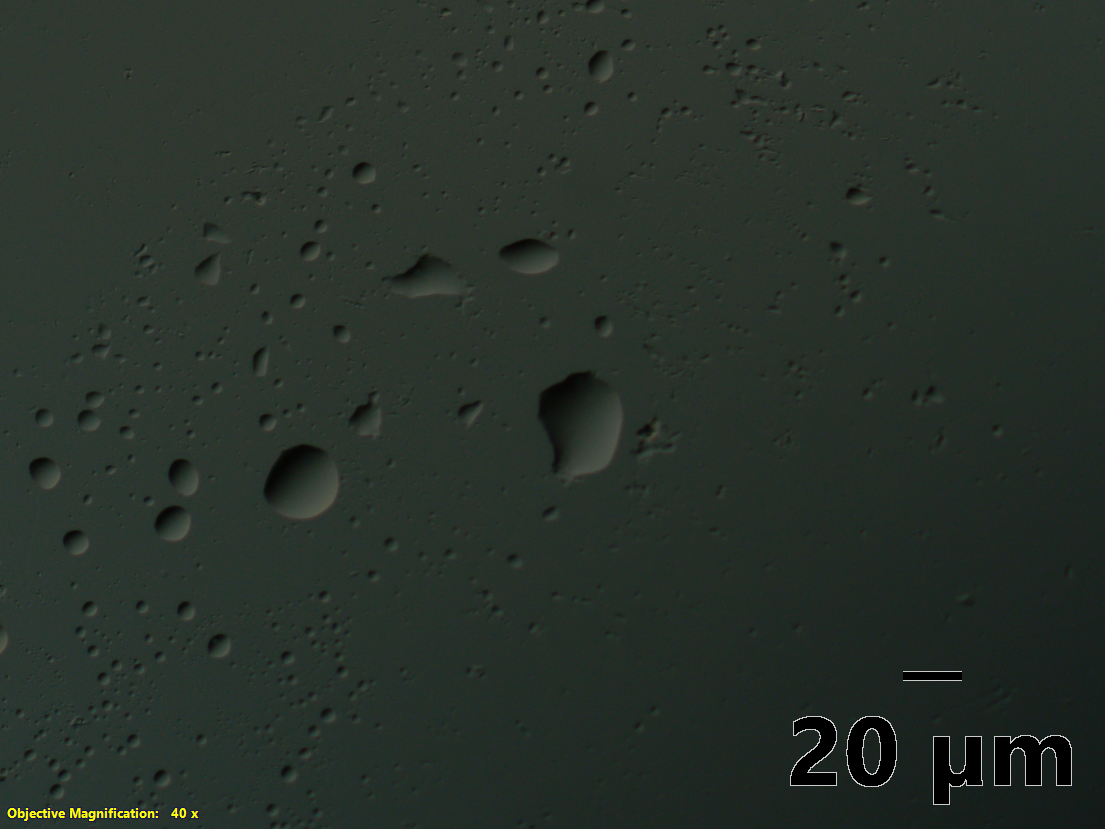

Supplement: Supplementary file 1 [file viruses-17-01473-s001.zip › Figure S21 3C RNA9.tif]

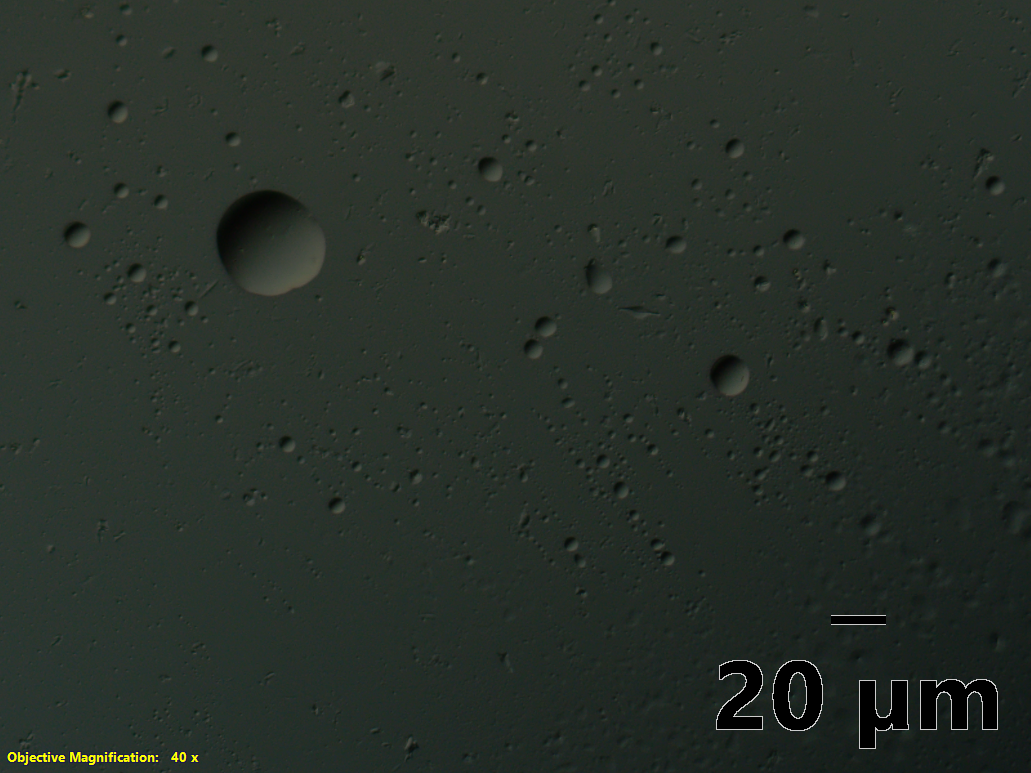

Supplement: Supplementary file 1 [file viruses-17-01473-s001.zip › Figure S22 3CD RNA10.tif]

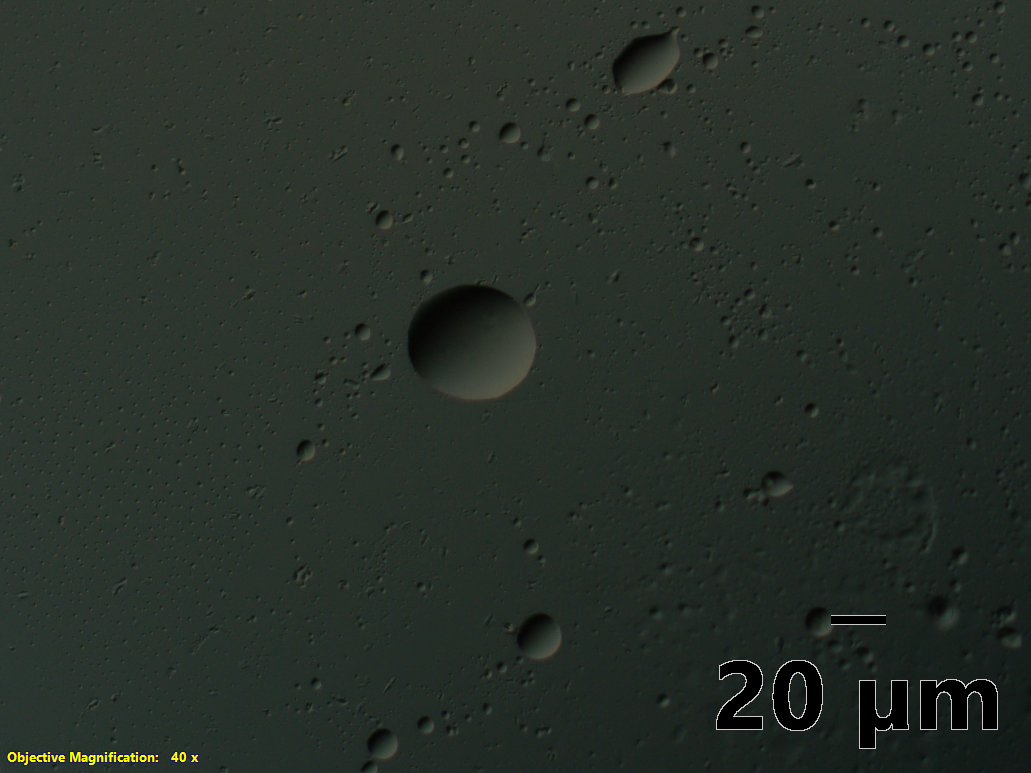

Supplement: Supplementary file 1 [file viruses-17-01473-s001.zip › Figure S22 3CD RNA11.tif]
